# Starch/Poly(butylene adipate-*co*-terephthalate) Blown Films Containing Quaternary Ammonium Salts and Ethylenediaminetetraacetic Acid for Food Preservation

**DOI:** 10.3390/foods15162797

**Published:** 2026-08-10

**Authors:** Shan Gao, Qiantong Wang, Yuchen Li, Yuanzhe Hou, Junjie Zhang, Yuntong Wu, Hanxue Hou

**Affiliations:** 1 College of Food Science and Engineering, Shandong Agricultural University, Tai’an 271018, China; 2023010160@sdau.edu.cn (S.G.);; 2 Engineering and Technology Center for Grain Processing in Shandong Province, Tai’an 271018, China; 3 National R&D Center for Beef Processing Technology, Tai’an 271018, China; 4 International Joint Research Lab (China and Greece) of Digital Transformation as an Enabler for Food Safety and Sustainability, Tai’an 271018, China; 5 Shandong Medicine Technician College, Tai’an 271000, China

**Keywords:** starch/PBAT film, quaternary ammonium salts, extrusion blowing, ethylenediaminetetraacetic acid, antibacterial capacity

## Abstract

In this study, quaternary ammonium salts (QAS) with different alkyl chain lengths (C12 and C18) were incorporated into starch/poly(butylene adipate-*co*-terephthalate) (PBAT) matrices with ethylenediaminetetraacetic acid (EDTA) to prepare blown films. The film with the combination of QAS and EDTA (D18D12E) achieved antibacterial rates of ≥99% against both *Staphylococcus aureus* and *Escherichia coli*, while maintaining cytocompatibility with HepG2 cells (viability ≥ 70%) and preliminary tobacco-growth compatibility. Compared with the control, the D18D12E film showed lower tensile strength (7.60 MPa) and higher oxygen (22.42 × 10^−13^ cm^3^·cm·cm^−2^·s^−1^·Pa^−1^) and carbon dioxide permeability coefficients (22.10 × 10^−13^ cm^3^·cm·cm^−2^·s^−1^·Pa^−1^). Its elongation at break (805.80%) and water vapor permeability (4.03 × 10^−11^ g·m·m^−2^·s^−1^·Pa^−1^) remained statistically comparable to those of the control. Furthermore, the D18D12E films delayed the visible deterioration of Chinese steamed bread and strawberries during 4 and 8 days of storage, respectively. These findings show that EDTA-assisted reduction in QAS loading can maintain antibacterial activity, but the associated mechanical and gas-barrier trade-offs require further formulation optimization.

## 1. Introduction

The globalization of fresh agricultural product storage, transportation, and distribution has driven an increasing demand for sustainable packaging materials capable of extending shelf life [1]. Among various biodegradable candidates, starch-based materials are considered promising due to their low cost, renewability, and biodegradability [2,3,4]. Our previous studies also showed that starch/poly(butylene adipate-*co*-terephthalate) (PBAT) composite was an attractive option, combining the economic advantages of starch with the excellent processability of PBAT [5,6,7]. However, such materials suffer from limited functional ability, restricting their effectiveness in preserving perishable foods [1].

Incorporation of active agents is a common strategy to extend the shelf life of perishable foods [8,9,10]. To date, many efforts have focused on integrating natural bioactive compounds, such as tea polyphenols, essential oil [11], ε-polylysine [12], nisin [13], anthocyanin [14], and quercetin [15], into starch/PBAT matrices. However, these natural additives are often associated with high costs and poor stability, and are particularly susceptible to degradation or deactivation during processing or storage [14]. In our previous work, we found that quaternary ammonium salts (QAS) were attractive cationic antibacterial agents [5], offering a cost-effective alternative with strong antibacterial activity [16]. Nevertheless, their application in food packaging systems remains challenging due to potential cytotoxicity and environmental concerns, especially for short-chain QAS with higher mobility [17]. Therefore, the application of QAS in food-contact packaging requires consideration of antibacterial function together with potential migration and exposure risks [16]. Regulation (EC) No 1935/2004 requires food-contact materials to avoid transferring constituents to food in quantities that could endanger human health or cause unacceptable changes in food. For plastic food-contact materials, Regulation (EU) No 10/2011 establishes an overall migration limit of 10 mg/dm^2^ and provides substance-specific restrictions where applicable.

A straightforward strategy to mitigate the cytotoxicity of QAS is to reduce their dosage. However, decreasing the concentration often leads to compromised antibacterial activity, particularly against Gram-negative bacteria. The unique outer membrane structure of Gram-negative bacteria, which contains lipopolysaccharides that act as an effective permeability barrier, can hinder the penetration of cationic agents [18]. As a result, QAS typically exhibit lower efficacy against Gram-negative strains compared with Gram-positive strains, making it difficult to achieve both high activity and low toxicity through dosage reduction alone [5]. To address this challenge, introducing auxiliary components that can enhance the susceptibility of Gram-negative bacteria while regulating QAS activity represents a promising strategy. Ethylenediaminetetraacetic acid (EDTA), a multicarboxylate compound, has the capacity to chelate divalent cations (e.g., Ca^2+^ and Mg^2+^) that stabilize the lipopolysaccharide structure [19]. This effect can disrupt the integrity of the bacterial outer membrane, thereby increasing the susceptibility of Gram-negative bacteria to antibacterial agents [13,20]. This mechanism provides a rationale for evaluating EDTA as an auxiliary component capable of maintaining antibacterial efficacy at a reduced QAS content. We hypothesized that combining EDTA with reduced amounts of D12 and D18 would maintain antibacterial efficacy, particularly against Gram-negative *Escherichia coli*, while providing a more favorable preliminary biological-compatibility profile than the QAS-only formulations.

Previous studies have investigated the influence of QAS alkyl-chain length in starch/PBAT blown films [5] and the performance of contact-killing cationic starch/PBAT films [18]. The present study is differentiated by evaluating an EDTA-assisted reduction in the total QAS content in an extrusion-blown starch/PBAT system and by jointly examining film structure, physicochemical properties, antibacterial performance, preliminary biological and migration assessments, and preservation performance. Specifically, starch/PBAT films containing didodecyl dimethyl ammonium chloride (D12), dioctadecyl dimethyl ammonium chloride (D18), and EDTA were fabricated by extrusion blowing. Their antibacterial performance was evaluated using *Escherichia coli* and *Staphylococcus aureus* as model bacterial strains. HepG2 cytocompatibility, tobacco-growth responses, and overall migration were also assessed to provide a preliminary evaluation of the biological compatibility and food-contact-related migration behavior of the films. The effects of the QAS-EDTA combination on the structures and physicochemical properties of the films were also systematically investigated. The findings support the potential of co-incorporating EDTA and QAS into starch/PBAT matrices as a formulation strategy for developing packaging materials with effective antibacterial performance and a favorable preliminary compatibility profile.

## 2. Materials and Methods

### 2.1. Materials

Hydroxypropyl distarch phosphate (HP-CF T0278), a modified cassava starch, was supplied by Puluoxing Starch Co., Ltd. (Hangzhou, China). According to the manufacturer, it contains 3.10% hydroxypropyl groups, along with 12.5% moisture, 0.26% lipid, 0.24% protein, and 0.23% ash. The amylose content (approximately 26%, *w*/*w*) was determined using a commercial amylose assay kit (BC4260, Beijing Solarbio Science and Technology Co., Ltd., Beijing, China). Poly(butylene adipate-co-terephthalate) (PBAT, BT1218) was obtained from Ruian Applied Bio-tech Co., Ltd. (Jining, China). The resin has a density of 1.21–1.24 g/cm^3^, a melting temperature range of 110–130 °C, and a melt flow index of 3–5 g/10 min (190 °C, 2.16 kg). Organically modified montmorillonite (Product Code: DK3) was obtained from Fenghong New Material Co., Ltd. (Anji, China). Glycerol, EDTA, and other analytical-grade reagents were purchased from Kaitong Chemical Reagent Co., Ltd. (Tianjin, China). Didodecyl dimethyl ammonium chloride (D12, purity 99%, ointment form) and dioctadecyl dimethyl ammonium chloride (D18, purity 97%, powder form) were supplied by Xiya Chemical Industry Co., Ltd. (Jinan, China). Strawberries were purchased from a local market in Tai’an, Shandong Province, China.

### 2.2. Film Preparation

Films were manufactured according to the established protocol in our prior study on starch/PBAT composites [6]. Starch, PBAT, glycerol, DK3, QAS, and EDTA were first premixed using a high-speed mixer (SHR-50A, Hongji Machinery Co., Ltd., Zhangjiagang, China) for 5 min and then melt-compounded in a twin-screw extruder (SHJ-20B, Giant Machinery Co., Ltd., Nanjing, China) equipped with alternating conveying and kneading elements. The screw had a diameter of 21.7 mm and a length-to-diameter ratio of 40. The temperature profile from the feeding zone to the die was set at 105–120–125–135–125–110 °C, with a screw speed of 150 rpm. The extrudates were air-cooled and pelletized into masterbatches. The obtained masterbatches were further processed into films using a single-screw extruder (SCM-50, Lianjiang Machinery Co., Ltd., Zhangjiagang, China). The extruder featured a screw diameter of 25 mm and a length-to-diameter ratio of 30, with a die diameter of 30 mm. The temperature profile was set at 110–130–140–145–135–125 °C from the feeding zone to the die, and the screw speed during the blowing process was maintained at 30 rpm.

This study used a non-factorial comparative formulation design. For physicochemical characterization, film formulation was the experimental factor with five levels: Control, D12, D18, D18D12, and D18D12E (Table 1). All formulations shared the same base matrix and processing conditions and differed only in their D12, D18, and EDTA compositions. The additive-free Control served as the material control. Table 1 reports quantities in grams per batch, with the combined starch and PBAT mass fixed at 1000 g; therefore, 30, 15, and 10 g corresponded to 3.0%, 1.5%, and 1.0% *w*/*w*, respectively. For the antibacterial assays only, three additional formulations were prepared under the same conditions: EDTA (3.0% *w*/*w* EDTA), D12E (1.5% *w*/*w* D12 and 1.5% *w*/*w* EDTA), and D18E (1.5% *w*/*w* D18 and 1.5% *w*/*w* EDTA).

### 2.3. Characterization of Physicochemical Properties

#### 2.3.1. Rheological Analysis

Rheological tests of the blends were conducted using a modular rotation rheometer (Model MCR 102, Anton-Paar, Graz, Austria) according to Gao et al. [6], where oscillatory shear measurements were performed from 0.1 to 100 rad/s at 145 °C, with a fixed strain of 0.1% to ensure linear viscoelastic response. One blend specimen per formulation was analyzed (*n* = 1), and the resulting rheological curves and fitted parameters were used for descriptive comparison.

#### 2.3.2. Fourier-Transform Infrared (FTIR) Spectroscopy

Fourier transform infrared (FTIR, model Nicolet iS10, Thermo Fisher Scientific, Waltham, MA, USA) spectroscopy spectra were recorded using an attenuated total reflectance (ATR) accessory over 4000–550 cm^−1^ at a resolution of 4 cm^−1^. FTIR measurements were performed on three independently prepared film specimens per formulation (*n* = 3), with 32 co-added scans collected for each specimen. One representative spectrum per formulation is presented for clarity.

#### 2.3.3. Low-Field Nuclear Magnetic Resonance (LF-NMR)

Proton spin-spin relaxation time (T_2_) curves of the films were recorded by a low-field nuclear magnetic resonance (LF-NMR) tester (model NMI20-015V-I, 23 MHz, Niumag Corp., Shanghai, China). Tests were conducted using the Carr–Purcell–Meiboom–Gill (CPMG) pulse sequence at 32 °C, with an echo number of 4000, an echo time of 0.50 ms, a scan number of 8, and a 2000 ms interval between successive scans. Measurements were performed on three independently prepared film specimens per formulation (*n* = 3). One representative spectrum per formulation is presented for clarity.

#### 2.3.4. X-Ray Diffraction (XRD)

The crystalline structure was characterized using an X-ray diffractometer (model Smartlab SE, Rigaku, Tokyo, Japan) equipped with an Ni-filtered Cu-K_α_ radiation. Data were recorded in the diffraction angle (2*θ*) of 10–40°. Relative crystallinity was calculated by using JADE software (version 6.5, Materials Data Inc., Livermore, CA, USA). One film specimen per formulation was analyzed (*n* = 1), and the resulting diffraction patterns and calculated relative crystallinity were interpreted descriptively.

#### 2.3.5. Thermogravimetric (TG) Analysis

Thermogravimetric (TG) analysis of the films was conducted using a TG analyzer (model TA-60, Shimadzu, Kyoto, Japan) from 25 to 600 °C at 10 °C/min under nitrogen flow (50 mL/min). One TG test run was performed for each formulation (*n* = 1), and the resulting thermal parameters were used for descriptive comparison only.

#### 2.3.6. Scanning Electron Microscopy (SEM)

Cross-sectional samples were prepared by cryo-fracturing in liquid nitrogen, then fixed to stub with double-side carbon tape and then sputter-coated with a layer of gold by an ion sputter coater. The observation was conducted by a scanning electron microscope (model Quanta FEG 250, FEI, Hillsboro, OR, USA) at 8.0 kV. Multiple fields were examined from one cross-sectional film specimen per formulation (*n* = 1 specimen), and representative micrographs are presented for qualitative morphological comparison.

#### 2.3.7. Mechanical Properties

Mechanical properties of the films were measured using a universal testing machine (model XLW, Labthink Instruments Co., Ltd., Jinan, China), according to the general procedure of GB/T 1040.3-2006 [21] for thin plastic sheeting. Rectangular specimens (100 mm × 15 mm) were clamped with an initial gauge length of 50 mm and stretched at a crosshead speed of 100 mm/min. Tensile strength (TS), elongation at break (EAB), and Young’s modulus (YM) were calculated to evaluate the mechanical performance. At least 5 test repetitions were performed for each film, and one film specimen constituted one experimental unit.

#### 2.3.8. Water Contact Angles

Dynamic contact angles were measured using a goniometer (model JC2000C1, Zhongchen Digital Technology Apparatus Co., Ltd., Shanghai, China) via the sessile drop method. A ~7 μL water droplet was placed on the film surface and recorded for 120 s at 0.3 s intervals. Contact angles were calculated using a contact angle measurement software (JC2000). At least 3 repetitions were performed for each film, and one film specimen constituted one experimental unit.

#### 2.3.9. Water-Vapor Permeability (WVP)

Water-vapor permeability (WVP) was measured using a PERME^TM^ W3/030 tester (Labthink Instruments) following the principle of GB/T 1037-2021 [22], where films (80 mm diameter) were tested at 38 °C and 90% RH after 4 h equilibration, with a 2 h weighing interval. WVP values of each sample were calculated from three separate measurements, and 3 film specimens constituted one experimental unit.

#### 2.3.10. Gas Permeability

The oxygen and carbon dioxide permeability coefficients (OP, CDP) of the films were determined using an auto differential-pressure gas permeation analyzer (model C106H, Labthink Instruments) following the principle of GB/T 1038.1-2022 [23]. Circular specimens (100 mm in diameter) were mounted in the test cells with an effective area of 38 cm^2^, and measurements were performed at 25 °C following a 2 h degassing step. Gas permselectivity was defined as the ratio of CDP to OP. Each value of the OP, CDP, and permselectivity ratio represented an average of triplicate samples, and one film specimen constituted one experimental unit.

#### 2.3.11. Light Barrier Properties

UV–visible absorption spectra of the starch/PBAT films (4 cm × 1 cm) were recorded from 200 to 800 nm using a spectrophotometer (model T700, Persee Universal Instrument Co., Ltd., Beijing, China) in accordance with GB/T 2410-2008 [24]. To account for differences in film thickness, the absorbance at each wavelength (*A_λ_*) was normalized to the corresponding film thickness (*x*, mm), and the wavelength-dependent opacity was expressed as Equation (1) [25].

(1) $$Opacity=\frac{A_{\lambda }\;}{x}$$

Measurements were performed on three independently prepared film specimens per formulation (*n* = 3). One representative curve per formulation is presented for clarity.

### 2.4. In Vitro Antibacterial Activity

In vitro antibacterial activity was evaluated using agar diffusion and plate counting methods according to the methods reported by GB/T 31402-2023 [26] and GB/T 39101-2020 [27] standards. Gram-positive *Staphylococcus aureus* (*S. aureus*, CMCC26003) and Gram-negative *Escherichia coli* (*E. coli*, CVCC1387) were used as model microorganisms. Before use, one loopful of each bacterial strain was inoculated into 20 mL of Luria–Bertani liquid culture medium (KGH2105-5.0 L, KeygenBioTECH, Nanjing, China) and cultured for 12 h. Bacterial suspensions were diluted with phosphate buffered saline (PBS) (P1034, Beijing Solarbio Science Technology Co., Ltd., Beijing, China) to inocula (approximately 1.0 × 10^6^ CFU/mL). Film samples (~40 μm thickness) were cut into circular disks (6 mm in diameter) and sterilized under UV irradiation prior to testing. All experiments for in vitro antibacterial tests were performed in triplicate. For the plate-counting assay, one independent film-inoculum system constituted one experimental unit. For the agar-diffusion assay, one independently inoculated agar plate constituted one experimental unit.

#### 2.4.1. Agar Diffusion Method

In total, 0.1 mL of bacterial inocula was evenly spread onto nutrient agar plates using sterile glass beads (CG5603, Beijing Coolaber Science and Technology Co., Ltd., Beijing, China) to ensure uniform distribution. Film disks were then placed on the agar surface at appropriate distances. After incubation at 37 °C for 24 h, inhibition zones were recorded and their diameters were measured using a digital vernier caliper.

#### 2.4.2. Plate Counting Method

Film disks were placed at the bottom of sterile centrifuge tubes, followed by the addition of 0.1 mL of bacterial inocula (1.0 × 10^6^ CFU/mL) to ensure direct contact. After incubation at 37 °C for 3 h, 5 mL PBS (DZ8023, UElandy, Suzhou, China) was added and the mixture was vortexed for 30 s. An aliquot (0.1 mL) was spread onto nutrient agar plates and incubated at 37 °C for 24 h. The viable bacterial concentration was calculated as Equation (2):

(2) $$N\;=\;\frac{C\;}{V\times d}$$

where *N* is the recovered viable count (CFU/mL), *C* is the number of colonies on the plate, *V* is the plated volume (mL), and *d* is the plated dilution fraction. The antibacterial rate and log reduction were calculated as follows:

(3) $$Antibacterial\;rate(\%)\;=\;\frac{N_{c}-N_{s}}{N_{c}}\times 100\%$$

(4) $$Log\;reduction={log}_{10}N_{c}-{log}_{10}N_{s}$$

where *N_c_* and *N_s_* are the viable bacterial counts recovered from the additive-free control and the antibacterial film, respectively. Notably, plates containing more than 200 colonies were considered too numerous to count and were reported as >200 colonies. Therefore, when the control plate contained >200 colonies and the corresponding D18D12E plate contained no detectable colonies, the antibacterial activity was conservatively expressed as >99% inhibition and >2-log reduction.

### 2.5. Preliminary Application in Strawberries and Chinese Steamed Bread

Fresh strawberries with uniform size, color, and no visible defects were selected and randomly divided into seven groups: unpackaged, and those packaged with polyethylene (PE), Control, D18, D12, D18D12, and D18D12E films. At each sampling time (days 2, 4, 6, and 8), three independently packaged fruits were evaluated for each treatment, with each fruit constituting one experimental unit (*n* = 3 per treatment per time point). Individual fruits were wrapped with the corresponding films, sealed, and placed in trays. All samples were stored at 25 °C for 8 days. The photographs at each time point show a representative fruit selected from the replicate samples. Weight loss was determined by measuring the initial and final weights using an analytical balance and expressed as a percentage of the initial weight.

Chinese steamed bread was prepared using commercial wheat flour (Zhongyu Wheat Flour, Zhongyu Food Co., Ltd., Binzhou, China) purchased from a local market. PE and representative prepared films were fabricated into packaging bags with a size of 25 cm × 15 cm. Each steamed bread sample was cut into four equal pieces. At each sampling time (days 2 and 4), three independently packaged steamed-bread samples were evaluated for each treatment, with two pieces of steamed bread constituting one experimental unit (*n* = 3 per treatment per time point). Samples without film packaging were used as the unpackaged group. All samples were stored at 23 °C, and their visual appearance was photographed on days 2 and 4 to record spoilage changes. The thickness and barrier properties of PE were measured as a thickness of 50 μm, a moisture permeability coefficient of 0.19 × 10^−11^ g·m·m^−2^·s^−1^·Pa^−1^ under 38 °C/90% RH, an O_2_ permeability coefficient of 2.43 × 10^−13^ cm^3^·cm·cm^−2^·s^−1^·Pa^−1^, and a CO_2_ permeability coefficient of 11.92 × 10^−13^ cm^3^·cm·cm^−2^·s^−1^·Pa^−1^. All experiments were performed in triplicate.

### 2.6. Preliminary Biological and Migration Assessments

#### 2.6.1. Tobacco-Growth Tests

To assess preliminary tobacco-growth responses, film fragments were mixed with a horticultural cultivation substrate at a film-to-soil mass ratio of 1:10 *w*/*w.* Each cultivation tray contained 5 g of film fragments and 50 g of substrate. The substrate was obtained from the Flower Demonstration Garden of the Teaching Base of Shandong Agricultural University and had an organic-matter content of ≥40%, a pH of 5.8–6.5, and an electrical conductivity of 1.0–1.5 mS·cm^−1^. Six treatments were evaluated: soil without film, Control, D12, D18, D18D12, and D18D12E. Soil without film served as the negative control, whereas soil containing the additive-free Control film served as the material control. Approximately 50 tobacco seeds were sown in each tray, with 3 independently prepared trays per treatment. Seedling emergence was visually recorded on day 5, and visible seedling development and morphology were documented on day 15.

#### 2.6.2. Cell-Compatibility Tests

HepG2 cells were obtained from Procell Life Science and Technology Co., Ltd. (Wuhan, China) and cryopreserved in serum-free cryopreservation medium (S9050S, UElandy, Suzhou, China). Film extracts were prepared by statically immersing individual film specimens (2 cm × 2 cm; total exposed surface area of approximately 8 cm^2^) in 5 mL of HepG2-specific culture medium (C110211, Beijing Solarbio Science and Technology Co., Ltd., Beijing, China) at room temperature (approximately 26 °C). The extraction ratio was therefore 1.6 cm^2^/mL. Separate extracts were collected after 24 h, filtered through 0.22 μm hydrophilic membrane filters, and used without dilution. After thawing, HepG2 cells were seeded in 96-well plates at 1.0 × 10^4^ cells per well. After cell attachment, the cells were exposed to the undiluted D12, D18, and D18D12E film extracts for 24 h. Cells treated with film-free HepG2-specific culture medium served as the negative control. For morphological observation, the cells were fixed with 4% paraformaldehyde (Beijing Coolaber Science and Technology Co., Ltd., Beijing, China) and stained with 0.5% crystal violet (KGE1202-100, KeygenBioTECH). After washing with PBS (KGL2206-500, KeygenBioTECH), images were obtained using an inverted fluorescence microscope (IX73P1F, Olympus, Japan). Cell viability was determined using a CCK-8 assay kit (Beijing Boxbio Science and Technology Co., Ltd., Beijing, China). All experiments were performed in triplicate. One independently treated culture well constituted one experimental unit.

#### 2.6.3. Overall Migration Tests

The overall migration of the films was determined according to the previously reported method with some modifications [28]. Briefly, D18D12E film specimens with a total exposed area of 2 dm^2^ were immersed in 200 mL of deionized water at 25 °C for 5 d, corresponding to an area-to-volume ratio of 1 dm^2^/L. Deionized water was selected as an exploratory aqueous simulant relevant to high-moisture, non-fatty foods. After incubation, the films were removed, and the migration solution was transferred to a pre-weighed evaporation dish, evaporated to dryness, and dried to constant weight. A blank simulant without film was treated under the same conditions for correction. The overall migration value was expressed as the mass of non-volatile residue per unit contact area (mg/dm^2^). Each test was performed in triplicate.

### 2.7. Statistical Analysis and Data Visualization

Experimental data were analyzed using IBM SPSS Statistics 29.0 (IBM Corp., Armonk, NY, USA). Before analysis, normality and homogeneity of variance were assessed using the Shapiro–Wilk and Levene’s tests, respectively. No significant violations of either assumption were detected (*p* > 0.05). Unless otherwise stated, differences among treatments were evaluated using one-way analysis of variance (ANOVA). Following a significant omnibus ANOVA, Duncan’s multiple range test was used for exploratory mean separation among samples.

Storage-related data were analyzed using a two-way linear mixed-effects model implemented with the MIXED procedure in SAS 9.0 (SAS Institute Inc., Cary, NC, USA). Packaging type, storage time, and their interaction were specified as fixed effects, whereas experimental replicate was included as a random effect. Following significant fixed effects, pairwise comparisons of least-squares means were performed using the pairwise difference (PDIFF) option. Statistical significance was set at *p* < 0.05 throughout.

All figures and graphs were prepared using Origin software (Version 2017, OriginLab Corp., Northampton, MA, USA) and Microsoft PowerPoint (Version 16.78.3, Microsoft Corp., Redmond, WA, USA). Principal component analysis (PCA) and correlation heatmap were visualized using ChiPlot (https://www.chiplot.online/) (2026). PCA was performed based on Euclidean distance after data normalization. Pearson’s correlation analysis was used to evaluate the relationships among different indicators, and the resulting correlation matrix was visualized as a heatmap.

## 3. Results and Discussion

### 3.1. Rheological Analysis

**Figure 1:**
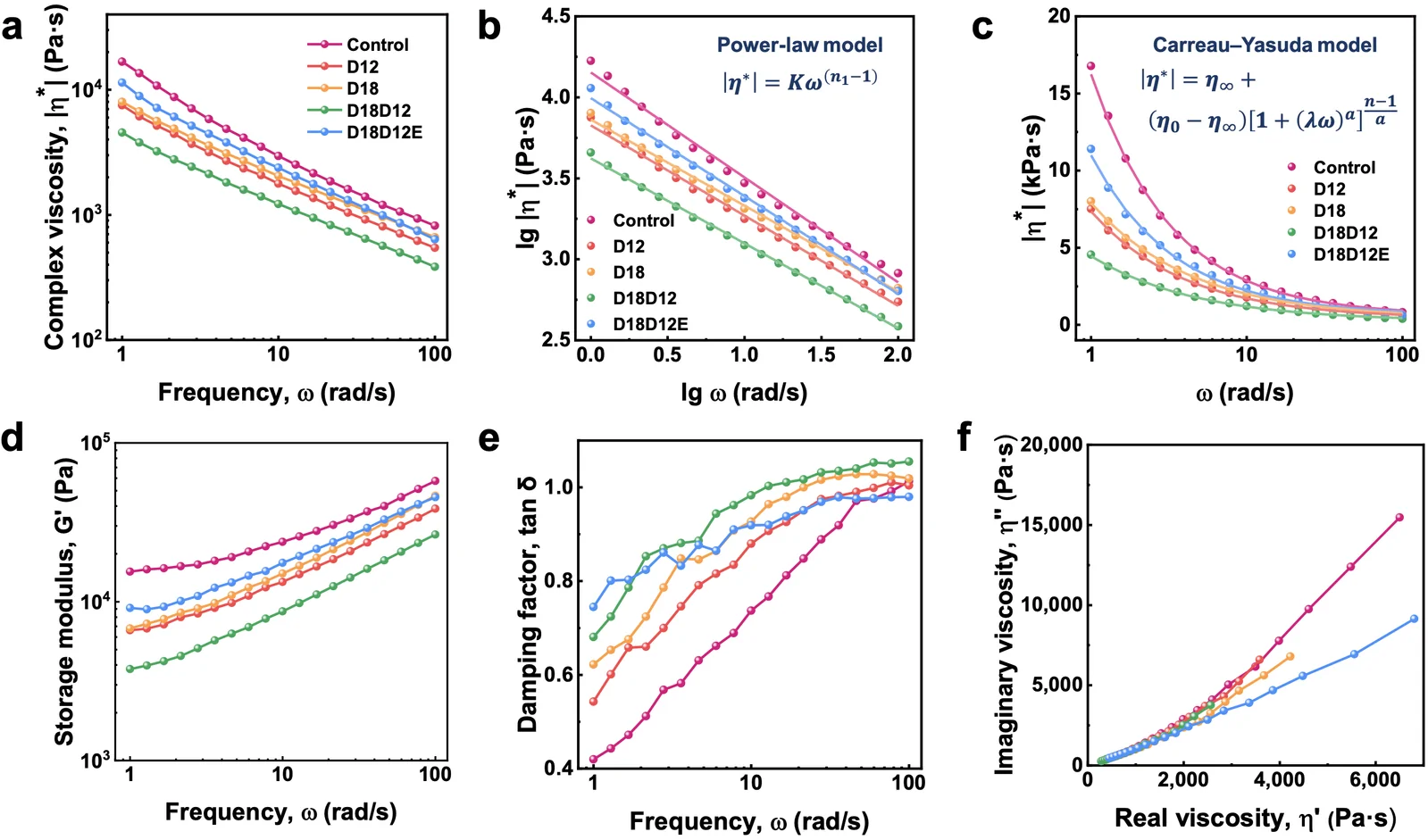
Rheological properties and model fitting of the blend with or without QAS and EDTA. (**a**) Complex viscosity (|η^*^|), and corresponding (**b**) Power-law and (**c**) Carreau–Yasuda model fitting of the frequency-dependent viscosity. (**d**) Storage modulus (G′), (**e**) damping factor (tan δ), and (**f**) Cole–cole plots of the blends.

Rheological properties of starch/PBAT blends were investigated to evaluate the influence of QASs and EDTA on the melt flow during processing. As shown in Figure 1a, all samples exhibited pronounced non-Newtonian flow, where the complex viscosity (|η^*^|) decreased continuously with increasing angular frequency. Such typical shear-thinning behavior reflected progressive alignment and disentanglement of polymer chains under shear [4,10]. Compared with the control, the incorporation of QASs reduced the viscosity across the entire frequency range, suggesting enhanced chain mobility and weakened intermolecular interactions [5,29]. The cationic head groups of the QASs interacted with starch through electrostatic attraction and hydrogen bonding, while the long hydrophobic alkyl chains acted as internal plasticizing segments [18]. These hydrophobic chains could increase the free volume within the starch domains, thereby facilitating molecular motion and reducing the melt viscosity of the starch/PBAT composites.

The Power-law and Carreau–Yasuda models well described the viscosity behavior, with R^2^ ≥ 0.99. Power-law model was described as Equation (5) and fitting results were presented in Figure 1b and Table 2.

(5) $$\left|\eta^{*}\right|=K\omega^{\left(n_{1}-1\right)}$$

Here, *K* represents the consistency factor, and *n*_1_ is the pseudoplastic index [30]. From the Power-law model, all samples exhibited pseudoplastic index (*n*_1_) < 1, confirming typical shear-thinning behavior. Notably, the incorporation of QAS increased *n*_1_ from 0.35 (Control) to 0.44–0.48, indicating a weakened shear-thinning effect. Meanwhile, the consistency index (*K*) decreased upon QAS addition, suggesting a reduction in system viscosity due to enhanced chain mobility and weakened intermolecular interactions [5,31]. Carreau–Yasuda model quantified the shear-induced relaxation behavior, as Equation (6).

(6) $$\left|\eta^{*}\right|=\eta_{\infty }+\left(\eta_{0}-\eta_{\infty }\right){\left[1+{\left(𝜆𝜔\right)}^{a}\right]}^{\frac{n-1}{a}}$$

Here, η_0_ and η_∞_ represent the zero-shear and infinite-shear viscosity, respectively. λ is the relaxation time [32]. The Carreau–Yasuda model (Figure 1c, Table 2.) revealed that the zero-shear viscosity (η_0_) decreased markedly after QAS incorporation, indicating reduced structural resistance at low shear force. The increased relaxation time (λ) indicated slower relaxation dynamics of the QAS-containing blends, reflecting a more flexible but less entangled chain structure [6,31]. Interestingly, the D18D12 sample showed the lowest η_0_ and highest λ. In contrast, the D18D12E sample exhibited a partial recovery in viscosity, suggesting that EDTA altered the relaxation behavior of the mixed-QAS formulation.

The storage modulus (G′) increased with frequency for all samples (Figure 1d), reflecting growing elastic response at shorter relaxation times [4]. The control displayed the highest G′, indicating a relatively stronger melt network in the starch/PBAT system. In contrast, the addition of D12 and D18 reduced G′, particularly in the D18D12 sample, implying that the amphiphilic surfactants weakened the intermolecular associations and promoted melt relaxation. Interestingly, the introduction of EDTA increased G′ compared with D18D12, suggesting partial reconstruction of molecular interactions within the blends [33]. The damping factor (tan δ) reflects the viscoelastic balance of the melts (Figure 1e). For all blends, tan δ increased with frequency, indicating a shift toward more viscous-dominant behavior (G″ > G′), which is mainly attributed to the limited relaxation ability of polymer chains under high-frequency oscillation. Notably, the control sample exhibited the largest variation in tan δ, suggesting a higher sensitivity to shear frequency. Consistently, the control sample showed the steepest increase in η″, while those with QASs displayed lower slopes in the Cole–cole plots (Figure 1f). These could be associated with the absence of QAS, as the incorporation of QAS can act as a plasticizing component, reducing molecular interactions and facilitating chain mobility, stabilizing the viscoelastic response.

### 3.2. ATR-FTIR Analysis

**Figure 2:**
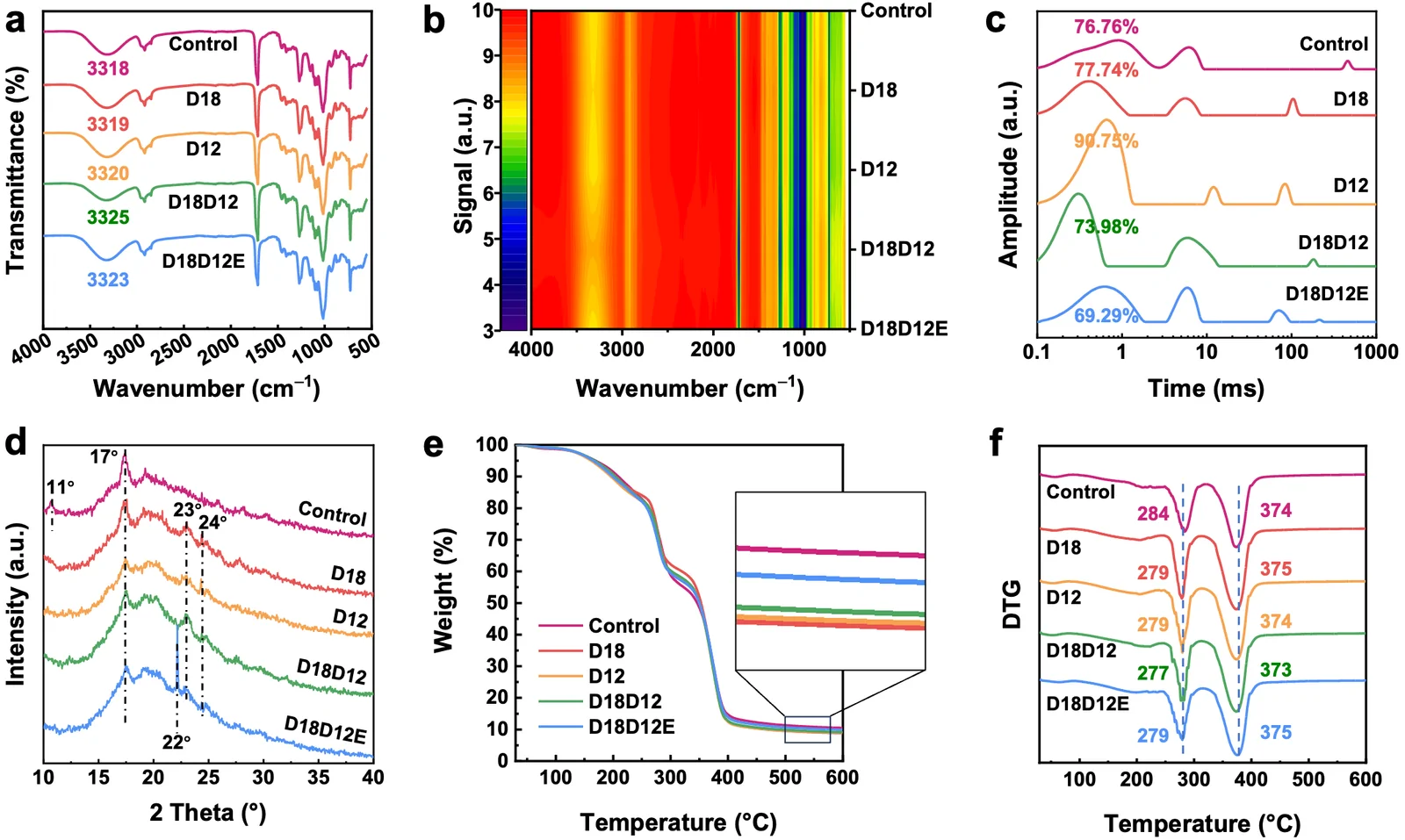
Structural characterization of starch/PBAT composite films containing QASs and EDTA. (**a**) FTIR spectra and (**b**) corresponding two-dimensional heatmap visualization of the spectra, (**c**) proton spin-spin relaxation time (T_2_) curves and corresponding bound water fractions, (**d**) XRD patterns, (**e**) TG curves, (**f**) first derivative thermogravimetric (DTG) curves.

The IR spectra of all samples exhibited similar characteristic bands (Figure 2a), and no new absorption bands were detected under the present measurement conditions. The broad band at 3289 cm^−1^ corresponded to O–H stretching vibration mode of hydroxyl groups (–OH). The strong band at approximately 1710 cm^−1^ and the bands near 1260 and 1100 cm^−1^ were mainly attributed to the ester C=O and C–O–C stretching vibrations of PBAT, respectively. The bands in the 1460–1370 cm^−1^ region were associated with C–H and CH_2_ deformation vibrations. In addition, the 1100–900 cm^−1^ fingerprint region included overlapping C–O and C–O–C vibrations from the anhydroglucose and glycosidic structures of starch, while the band near 800–700 cm^−1^ was associated with the out-of-plane C–H vibration of the aromatic structure of PBAT [5,13,34]. Compared with the control film, the O–H stretching band around 3200–3400 cm^−1^ exhibited a slight blue shift after the incorporation of D12, D18, and EDTA, accompanied by noticeable changes in signal intensity (Figure 2b). The cationic head groups of QASs may associate with starch hydroxyl groups, and the multiple carboxyl groups in EDTA may also participate in hydrogen bonding with hydroxyl groups in starch [5,13]. Notably, compared with the D18D12 film, the introduction of EDTA (D18D12E film) resulted in a red shift (2 cm^−1^) of the O–H stretching band. According to the harmonic-oscillator model, the peak frequency is negatively correlated with the molecular interaction. This suggested that stronger hydrogen-bonding interactions might be formed in the composite system [20]. Compared with QAS molecules, EDTA possesses multiple carboxyl groups and a relatively small molecular weight, which might provide more accessible hydrogen-bonding sites per mole [13]. Except for the broad O–H band, other characteristic bands showed no discernible shifts among the film formulations. This was because D12, D18, and EDTA were incorporated at relatively low levels compared with the starch/PBAT matrix. A similar phenomenon has been reported for extrusion-blown starch-based films containing a low concentration of an active additive [35].

### 3.3. LF-NMR Analysis

LF-NMR curves in Figure 2c revealed the molecular mobility and water distribution within the starch/PBAT films. All samples exhibited multiple relaxation components, indicating that water/proton populations existed in different binding states within the composite matrix. The short-relaxation component was assigned to tightly bound water associated with starch hydroxyl groups, glycerol, and restricted polymer domains, whereas the longer-relaxation components reflected weakly bound or more mobile water populations [14,36]. The control film showed a bound-water fraction of 76.76%. After the addition of D18, this value slightly increased to 77.74%, suggesting that D18 did not markedly change the water-binding state of the matrix. In contrast, the D12 film showed the highest bound-water fraction (90.75%), accompanied by a more dominant short-relaxation signal. This result indicates that D12 promoted stronger water immobilization in the film matrix, probably because its shorter alkyl chains provided a relatively weaker hydrophobic shielding effect and allowed greater interaction among QAS head groups, starch hydroxyl groups, glycerol, and water molecules [5,29]. When D18 and D12 were incorporated together, the bound-water fraction decreased to 73.98%, lower than that of the control and the single-QAS films. This decrease suggests that the coexistence of long and short alkyl chains altered the hydrophilic/hydrophobic microenvironment and weakened the continuous water-binding network in the starch-rich domains [29]. After EDTA was further introduced, the D18D12E film showed the lowest bound-water fraction (69.29%) and a relatively stronger contribution from longer-relaxation components. This result suggests that EDTA did not merely enhance water immobilization through hydrogen bonding. Instead, it likely competed with water molecules for starch hydroxyl sites and interacted with QAS molecules, thereby altering the distribution and binding state of water within the polymer matrix [13,20].

### 3.4. XRD Analysis

The crystalline structure of the films was presented in Figure 2d. The control exhibited several diffraction peaks located at approximately 11°, 17°, and 19°, which were attributed to the crystalline structures associated with starch and PBAT phases [13,37]. Concurrently, diffraction signals above 25° are generally associated with oriented PBAT-rich structures generated during film blowing [6]. After QAS incorporation, the relative crystallinity increased from 20.28% for the control to 22.75% for D12 film and 30.27% for D18 film. A diffraction peak appeared around 23°, indicating altered molecular packing during stretching and cooling [5,6,38]. The diffraction peak around 24°, which is associated with the recrystallization of starch, also increased in intensity. The interaction between QAS and starch could partially shield the starch-starch hydrogen-bonding interactions within starch domains, thereby promoting the rearrangement of starch chains to form B-type crystals [36]. This interpretation is consistent with the FTIR results, i.e., QASs may modify the existing hydrogen-bonding environment to change chain rearrangement. The D18 film showed stronger diffraction features than D12, showing higher relative crystallinity measured for D18. This difference may be related to the greater affinity of the longer hydrophobic alkyl chains of D18 for PBAT-rich domains, which could reduce interfacial constraints on molecular rearrangement [5,39]. When both QASs were incorporated, D18D12 exhibited a relative crystallinity of 24.89%, intermediate between those of D12 (22.75%) and D18 (30.27%). However, residual starch granules were observed in the SEM images of D18D12. This apparent discrepancy may arise from the different spatial scales and structural information represented by the two techniques. After the further introduction of EDTA, a distinct diffraction feature appeared around 22°, accompanied by a slight increase in relative crystallinity from 24.89% for D18D12 to 25.47% for D18D12E. These results likely occurred because the multiple carboxyl groups of EDTA can form hydrogen bonds with starch hydroxyl groups, thereby promoting molecular packing [13,20].

### 3.5. Thermal Stability

The thermal stability of the starch/PBAT composite films was evaluated by TG assessments, as presented in Figure 2e,f. The first derivative thermogravimetric (DTG) curves show two main peaks, corresponding to the thermal decomposition of starch (low-temperature) and PBAT (high-temperature), respectively [6]. Compared with the control, the maximum degradation temperature of the starch shifted slightly toward lower temperatures in the films with QASs and EDTA. This phenomenon could be attributed to two possible factors. First, the interaction between QAS molecules and starch hydroxyl groups might inhibit the reconstruction of intermolecular hydrogen bonding during the cooling stage of the film-blowing process [2]. As a result, the starch domains became thermally less stable and more susceptible to thermal decomposition due to the enhanced chain mobility [39]. Second, QAS molecules generally exhibit relatively low thermal decomposition temperatures. Their early thermal degradation during heating may generate volatile products that partially disrupt the polymer network, thereby facilitating the subsequent thermal decomposition of the starch phase [2,5,8]. In contrast, the high-temperature degradation peak corresponding to PBAT remained relatively stable, suggesting that the PBAT-rich phase retained its intrinsic thermal stability within the composites. Although the temperature at 50% weight loss (T_d50_) increased after the incorporation of QASs and EDTA, the modified films exhibited lower T_d5_ (5% weight loss) and T_d10_ (10% weight loss) values and a lower final residue than the control (Table S1). This behavior suggests that the small-molecule additives promoted the initial degradation of the films, whereas their interactions with the polymer matrix may have delayed decomposition at later stages. Meanwhile, QASs and EDTA were likely converted into volatile products during thermal degradation, thereby reducing carbonaceous residue formation [5,40].

### 3.6. Morphology

**Figure 3:**
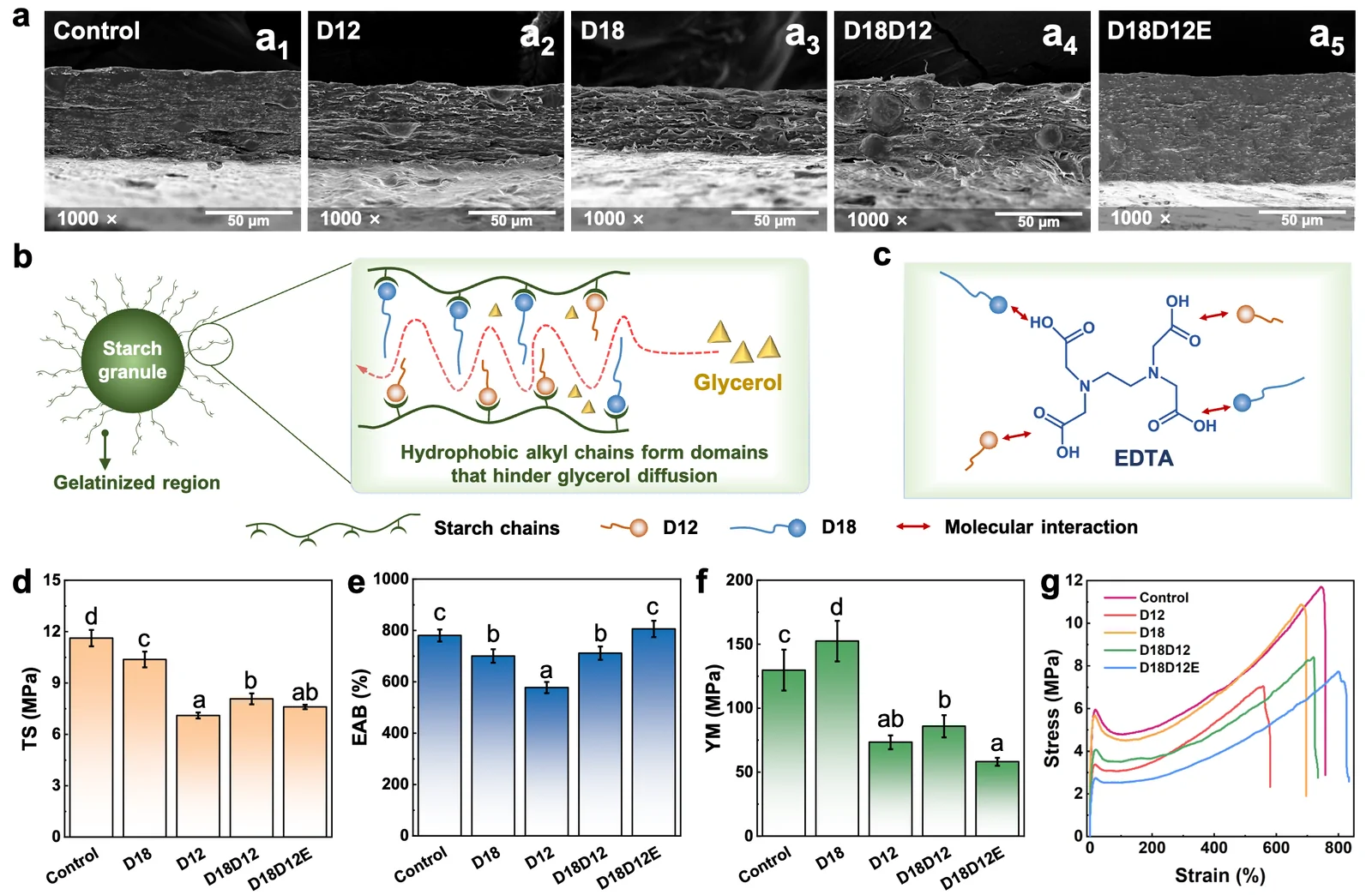
(**a**) Cross-sectional SEM images of the control, D12, D18, D18D12, and D18D12E films (1000×). (**b**) Schematic illustration of the anti-plasticization mechanism induced by QASs. (**c**) Proposed molecular interactions among D12, D18, and EDTA. (**d**) TS, (**e**) EAB, and (**f**) YM values of the films. (**g**) Representative stress–strain curves of the films. Results are quoted as means ± standard deviation. a–d: Different letters within the same panel indicate significant differences among the samples (*p* < 0.05).

Compared with the control, the films with QASs exhibited sheet-like protrusions that appeared to be pulled out from the fracture section (Figure 3a). This suggested a more ductile cryo-fracture behavior, contributing to the formation of lamellar-like fracture structures [5]. Several granular features were also observed in the D18D12 film. Magnified images of these features are provided in Figure S1. Microcracks were visible adjacent to the granular structures, as indicated by the yellow arrows. Similar microcracks were previously associated with starch granules in our previous study [6,35]. The hydroxypropyl distarch phosphate used in this study is a modified starch that still retains part of the native granular structure, with most modifications occurring on the granule surface [41]. During extrusion, the surface of starch granules underwent gelatinization, allowing plasticizers to penetrate and promoting the granular disruption [42]. When both D18 and D12 are present, the cationic head groups of QAS molecules could interact with starch hydroxyl groups. Their hydrophobic alkyl chains with different lengths might form a complex hydrophobic environment around starch domains, which could hinder the diffusion of small plasticizing molecules such as glycerol (Figure 3b). As a result, the gelatinization of starch granules might be partially suppressed, leading to the presence of residual granular structures.

This result differs from the observations reported by Fang et al. [2], who found that grafting hydrophobic styrene side chains onto starch enhanced glycerol plasticization. In this work, hydrophobic chains originated from free QAS molecules that interacted physically with starch chains. The grafted hydrophobic chains disrupted the internal hydrogen-bonding network of starch and facilitated glycerol penetration, while the free hydrophobic chains in the current system instead may have created localized hydrophobic environments that restricted plasticizer diffusion. After the introduction of EDTA, these granular features were no longer observed. The absence of these granular features after EDTA addition may reflect a change in the local organization of the QAS-containing domains. We hypothesize that EDTA–QAS associations, as schematically illustrated in Figure 3c, could contribute to this reorganization and potentially promote glycerol penetration into the starch-rich regions. However, these proposed interactions require further direct molecular-level verification.

### 3.7. Mechanical Properties

The mechanical properties of the starch/PBAT composite films are listed in Figure 3d–f. The QAS-containing films without EDTA generally showed lower TS and EAB than the control. This reduction may be associated with the effects of QAS molecules, whose hydrophobic alkyl chains may alter chain packing and weaken intermolecular interactions within starch domains, thereby reducing the load-bearing capability of the polymer network [16,43]. Notably, the “free” hydrophobic chains introduced by QAS molecules seem to differ from the grafted hydrophobic chains reported by Fang et al. [2]. The hydrophobic moieties, covalently attached to the pyranose units, could introduce additional chain entanglements to restrict molecular mobility. Conversely, the hydrophobic chains associated with QAS molecules did not function as reinforcing elements. This behavior was more pronounced in the films containing D12 (D12, D18D12, D18D12E), which exhibited earlier yielding followed by a rapid transition into a strain-softening region (Figure 3g), indicating that the system tended to dissipate stress through plastic deformation rather than elastic response. The incorporation of QAS alone reduced the flexibility of the films compared with the control. This was consistent with the presence of granular structures in their cross-sectional morphology. These residual starch granules might weaken the interfacial adhesion between starch and PBAT phases and lead to heterogeneous stress distribution, thereby causing premature failure of the load-bearing network [44]. Interestingly, the incorporation of EDTA significantly restored EAB relative to the D18D12 film. Nevertheless, compared with the control, D18D12E showed significantly lower TS (7.60 vs. 11.62 MPa) and YM (58.11 vs. 129.72 MPa), and its numerically higher EAB (805.80 vs. 780.47%) was not statistically significant. Thus, D18D12E retained control-level extensibility but exhibited reduced strength and stiffness. This pattern may indicate that EDTA promoted more uniform plastic deformation; however, this change did not restore the load-bearing capacity of the matrix [13,20].

### 3.8. Barrier Properties

**Figure 4:**
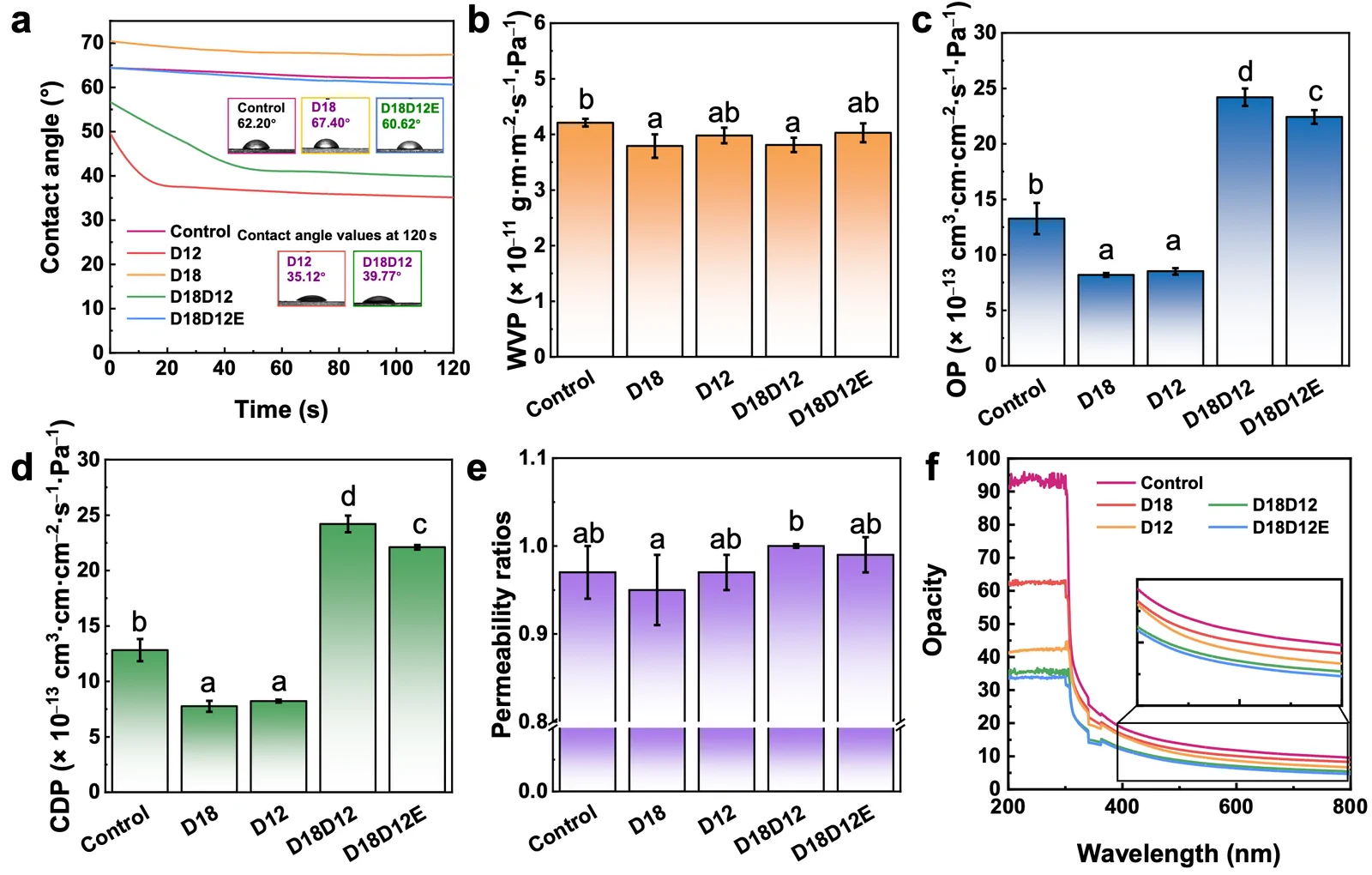
(**a**) Dynamic water contact angle of the films measured over 120 s and the water droplet images at 120 s. (**b**) WVP, (**c**) OP, (**d**) CDP, and (**e**) gas permeability ratio values of the films. (**f**) UV–vis spectra of the films over the wavelength range of 200–800 nm. Results are quoted as means ± standard deviation. a–d: Different letters within the same panel indicate significant differences among the samples (*p* < 0.05).

The moisture barrier properties of the starch/PBAT films are listed in Figure 4. The introduction of QAS formulations reduced the WVP values compared with the control. Among the films, the D18 film exhibited the minimum WVP value, which may be related to the ability of its longer hydrophobic alkyl chains to form discontinuities in the hydrophilic phase, hindering the diffusion of water molecules [5]. The contact angle results further support this observation. The D18 film showed the maximum initial contact angle (70.43°), indicating a more hydrophobic surface compared with the control (64.38°). In contrast, the D12 film exhibited a much lower initial contact angle (49.70°), suggesting increased surface wettability. Notably, the D18D12 film maintained relatively low WVP while showing moderate contact angles. This suggested that the coexistence of QAS molecules might form hydrophobic microdomains that partially restrict water diffusion. Moreover, the reduced plasticization of starch chains decreased the exposure of hydrophilic hydroxyl groups, thereby limiting water adsorption and diffusion [14,45]. The presence of EDTA slightly increased the WVP values (*p* > 0.05), which was related to the disruption of hydrophobic aggregation and additional hydrophilic carboxyl groups within the matrices [44].

For fresh fruits and vegetables, packaging films with appropriate gas barrier properties are considered essential for regulating respiration rates [1]. As presented in Figure 4c–e, compared with the control, the incorporation of QAS alone (D18, D12) significantly reduced both O_2_ permeability (OP) and CO_2_ permeability (CDP). This improvement could be attributed to the presence of hydrophobic alkyl chains in QAS molecules, which enhanced the compactness of the matrix to create more tortuous pathways for gas diffusion. However, when D18 and D12 were simultaneously introduced, the OP and CDP values increased markedly (D18D12, *p* < 0.05). This phenomenon was likely associated with the partial suppression of starch gelatinization and the presence of residual granular structures observed in SEM images, which introduced micro-voids or structural heterogeneity within the composite matrix and facilitated gas diffusion [9]. After EDTA incorporation, gas permeability decreased significantly compared with that of the D18D12 film (*p* < 0.05). However, the gas-barrier performance remained inferior to that of the control, possibly because the simultaneous presence of D18 and D12 still created residual free volume and hydrophilic transport pathways within the polymer matrix. In terms of permeability ratios (CO_2_/O_2_), all films exhibited values close to unity, indicating similar diffusion behaviors of CO_2_ and O_2_ in the composite matrix.

UV–vis spectra in Figure 4f reflect the effects of QASs and EDTA on the optical properties of the starch/PBAT films. After the incorporation of QASs, the opacity of the films decreased significantly, particularly for the D18 and D12 samples. This change suggested that the addition of QAS molecules improved the dispersion of starch domains and reduced microstructural heterogeneity, thereby decreasing light scattering within the films [3]. Interestingly, the D18D12 film showed slightly higher opacity than the single-QAS systems. This was attributed to the presence of residual granules that enhanced light scattering within the film matrix [46]. After introducing EDTA, the opacity decreased again, which was likely attributed to the fact that EDTA promoted a more uniform molecular arrangement by weakening hydrophobic aggregation among QAS molecules [13].

### 3.9. In Vitro Antibacterial Activity

Antibacterial activity of the films was assessed by both plate-counting and agar-diffusion methods. Specifically, the plate-counting assay was used to evaluate contact-mediated antibacterial activity, whereas the agar-diffusion assay was used only to assess the diffusion of antibacterial components through the agar.

**Figure 5:**
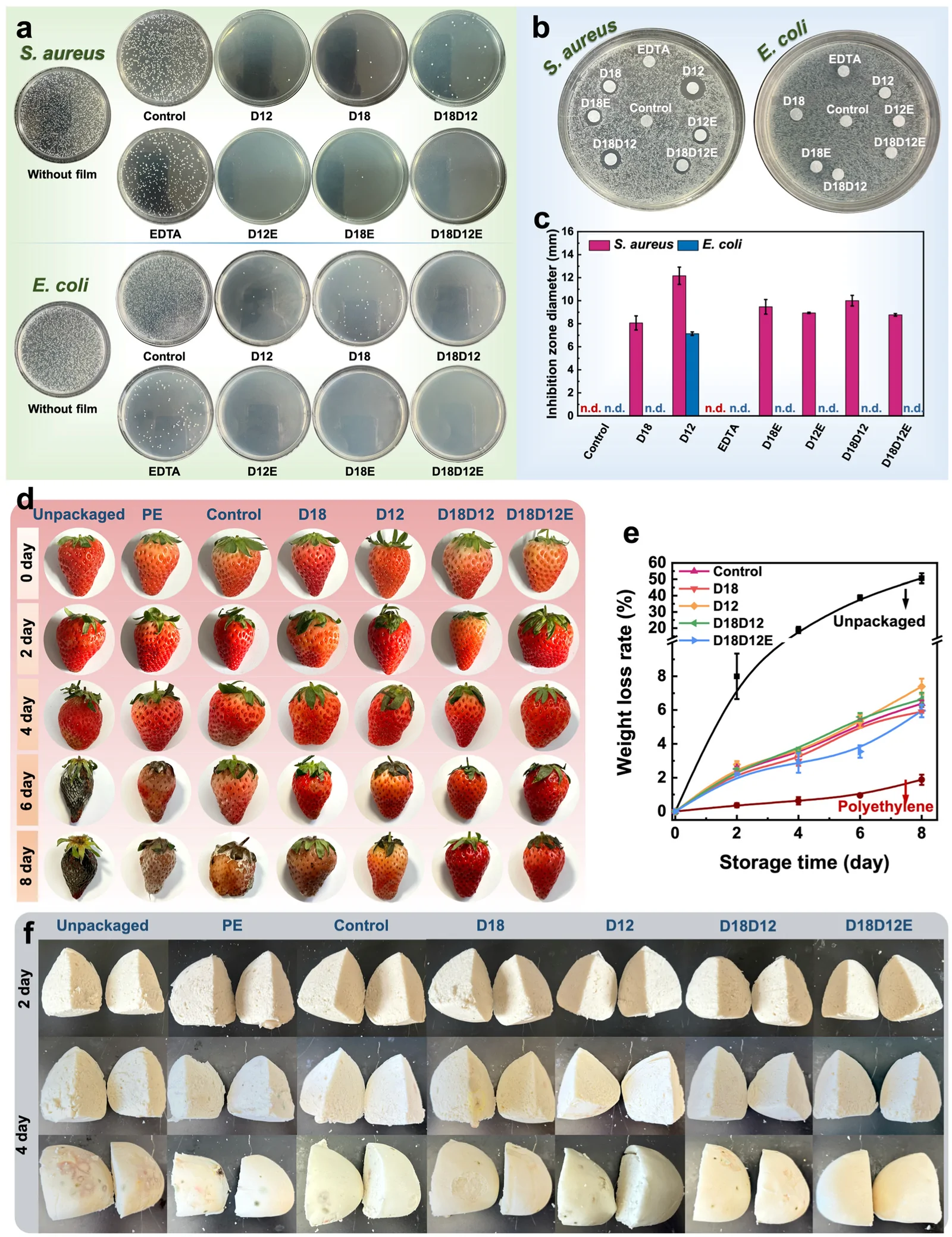
Antibacterial activity of the films against *S. aureus* and *E. coli*: (**a**) Representative images for plate colony counting assessments. (**b**) Agar diffusion assay of the films and corresponding (**c**) quantitative analysis of inhibition zone diameter (mm) for different samples. (**d**) Changes in appearance and (**e**) weight loss of the unpackaged strawberries and those packaged with different films during storage. (**f**) Representative appearance of Chinese steamed bread packaged with different films after 2 and 4 days of storage.

In the plate-counting assay (Figure 5a), the groups without films or with the control film showed extensive bacterial growth with over 200 colonies (>2.0 × 10^3^ CFU/mL in the PBS-recovered suspensions), whereas the QAS-containing films reduced the number of recoverable colonies. The positively charged quaternary ammonium groups can interact electrostatically with the negatively charged bacterial cell, leading to membrane disruption and leakage of intracellular components [18]. The films containing EDTA alone also reduced the number of countable bacterial colonies. It has been reported that EDTA can chelate divalent cations present in bacterial cell walls, thereby increasing the permeability of the cell envelope and weakening the structural integrity of bacterial membranes [13,19]. Notably, no colonies were detected in any D18D12E replicate for either bacterial strain (<10 CFU/mL). Relative to the control groups, D18D12E therefore achieved conservative lower bounds of >99% inhibition and > 2-log reduction.

The agar diffusion assay further revealed differences in antibacterial diffusion ability among the films. Notably, the D12 film exhibited the largest inhibition zone, consistent with our previous findings [5]. This phenomenon was associated with the increased hydrophilicity of the D12-containing film, which favored the release and diffusion of antibacterial components into the agar medium [47]. Interestingly, the films containing both QAS and EDTA (D12E, D18E, D18D12E) exhibited smaller inhibition zones despite maintaining strong antibacterial performance in the plate colony assay, compared with the D12 film. Together with the plate-counting results, the smaller inhibition zones suggested a greater contribution of contact-mediated killing than diffusion. However, the *n.d.* inhibition zones against *E. coli* indicated that no detectable diffusion zone formed under the assay conditions. This limited diffusional activity may restrict the use of the films in applications where antibacterial agents are required to migrate into the surrounding food or medium.

### 3.10. Preliminary Application in Strawberries and Chinese Steamed Bread

The packaging-application capacity of the starch/PBAT composite films was evaluated using fresh strawberries and Chinese steamed bread, as shown in Figure 5d–f. Rapid deterioration was observed in the unpackaged strawberries, with noticeable wilting at the 4th day, followed by severe fungal growth and tissue collapse after 6–8 days, accompanied by the highest weight loss (*p* < 0.05, Table S2). Such deterioration is consistent with the high perishability of strawberries, where respiration, transpiration, and microbial growth jointly drive quality loss during storage [48]. In contrast, all film-packaged samples showed less visible deterioration and reduced water loss. The PE group exhibited the lowest weight loss, but obvious quality deterioration was still observed, probably due to the moisture accumulation within the package. The control film, despite its relatively high moisture permeability, showed visible mold development on the 6th day and complete fruit decay on the 8th day. Similarly, the D18 film failed to maintain strawberry quality throughout storage and became inedible on the 8th day. Its relatively strong water barrier property may have resulted in higher internal humidity, which could accelerate respiration and microbial growth. In contrast, films containing D12 showed less visible spoilage, with D18D12 and D18D12E films maintaining good appearance by the end of storage. The observed differences in appearance may be associated with combined effects of antimicrobial activity and gas-exchange ability [49,50,51].

As a traditional staple food in China, Chinese steamed bread is susceptible to microbial spoilage because of its high-moisture content. No obvious spoilage was observed in any group after 2 days of storage. By the 4th day, visible spoilage (reddish spots) appeared on the tray-contact surface of the unpackaged sample, with a desiccated appearance. In comparison, the packaged samples generally appeared less desiccated, suggesting that packaging limited visible dehydration. However, red and black mold spots were still observed in the PE-packaged sample, associated with high-contact humidity caused by moisture retention inside the package. Similar mold growth was also found in the D18- and D18D12-packaged samples, probably because their strong moisture-barrier properties favored local moisture accumulation. By contrast, the D18D12E film maintained the best appearance of Chinese steamed bread during storage.

Because the storage tests relied mainly on visual assessment, these findings should be considered preliminary. Future studies should include food-associated microbial counts and instrumental measurements of color, texture, moisture content, and pH, together with weight-loss measurements for both food models. Under the tested conditions, strawberries packaged with D18D12E showed relatively limited visible deterioration over 8 days, while Chinese steamed bread packaged with D18D12E showed the least visible deterioration among the tested groups after 4 days. It also achieved antibacterial rates of at least 99% against both strains while using one-third less total QASs, making it a promising formulation for further study.

### 3.11. Preliminary Cytocompatibility, Tobacco-Growth Response, and Overall Migration

**Figure 6:**
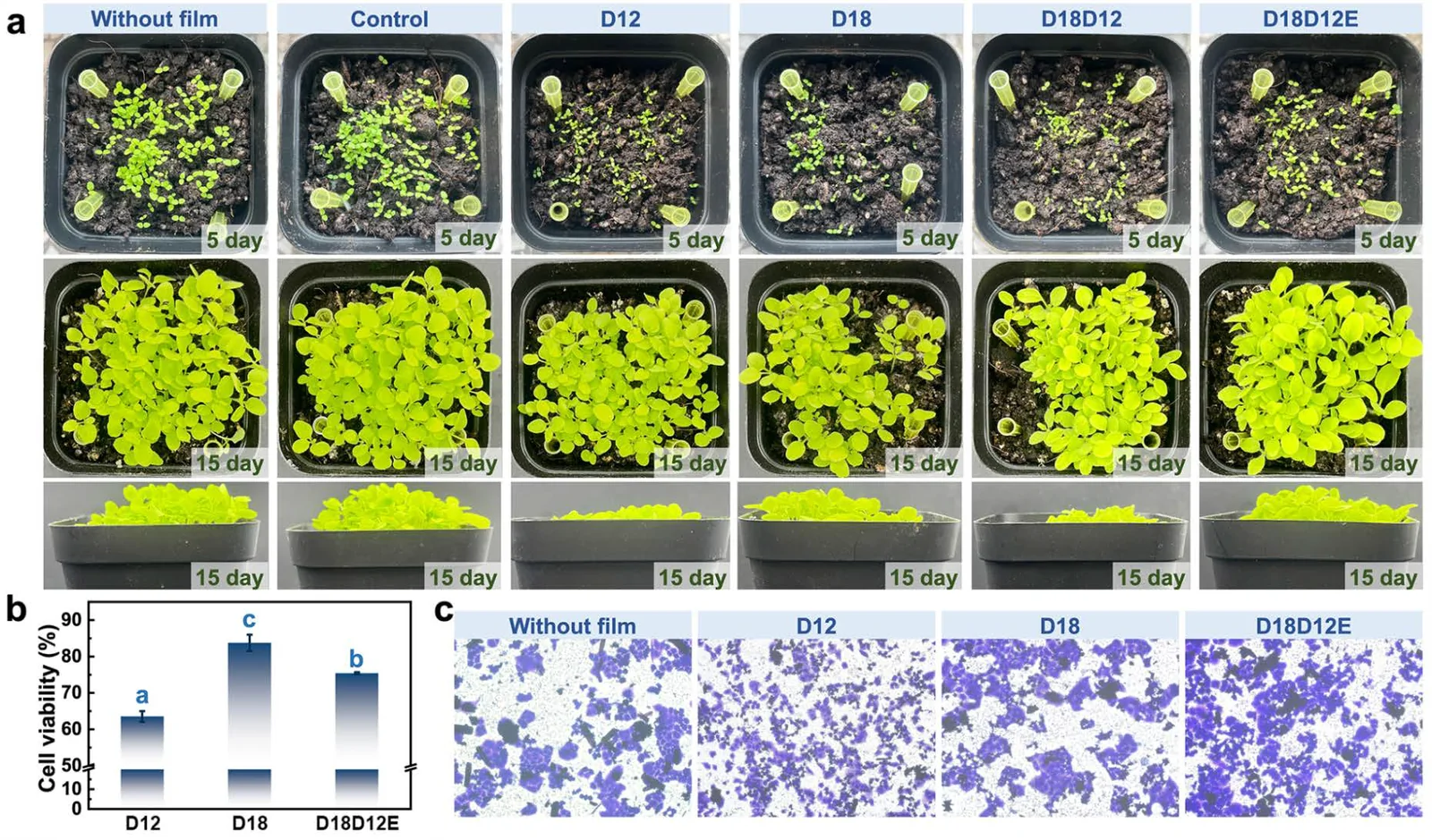
Preliminary cytocompatibility and tobacco-growth responses. (**a**) Growth behavior and appearance of seedlings in the soil without or with films. (**b**) Viability of the HepG2 cells treated with the aqueous extracts of D12, D18, and D18D12E films after 24 h of immersion, and (**c**) representative micrographs of the treated HepG2 cells stained with crystal violet.

As shown in Figure 6, tobacco seeds germinated in all treatment groups, and no complete inhibition of early seedling development was visually observed [18,52]. However, a slight retardation in plant growth was observed in the presence of QAS-containing films, particularly for the D12 group. This suggested that short-chain quaternary ammonium salts imposed a certain level of stress on early plant development. Consistent trends were observed in the cytocompatibility evaluation. The cells treated with the D12 film extract appeared more dispersed with reduced cell–cell contacts, which was likely attributed to the stronger membrane-disruptive capability of short alkyl chains, leading to weakened intercellular interactions [17]. In contrast, the cells exposed to the D18 film maintained a well-spread morphology with clear intercellular adhesion and clustering behavior, comparable to the control group. Notably, the D18D12E film exhibited improved biocompatibility compared to the D12 film, with cell viability above 70%, a level generally considered acceptable for preliminary cytocompatibility evaluation [53]. In addition, the plant height in this group was closer to that of the film-free and control groups. This could be attributed to the introduction of EDTA, which modulated the availability of QAS through interactions, thereby mitigating its cytotoxic effects [13,20]. Regarding migration behavior, the overall migration of D18D12E was 8.18 mg/dm^2^ under aqueous condition, below the specified limit of 10 mg/dm^2^ for plastic food-contact materials. Nevertheless, these preliminary assessments were limited by the use of a single cell model and by the limited duration, controls, and endpoints of the tobacco-growth assay. Before practical food-contact application, more rigorous evaluation will therefore be required, including multiple cell models, realistic exposure scenarios, longer-term degradation studies, and migration testing using food-category-appropriate simulants with compound-specific quantification of D12, D18, and EDTA.

### 3.12. Correlation Analyses

**Figure 7:**
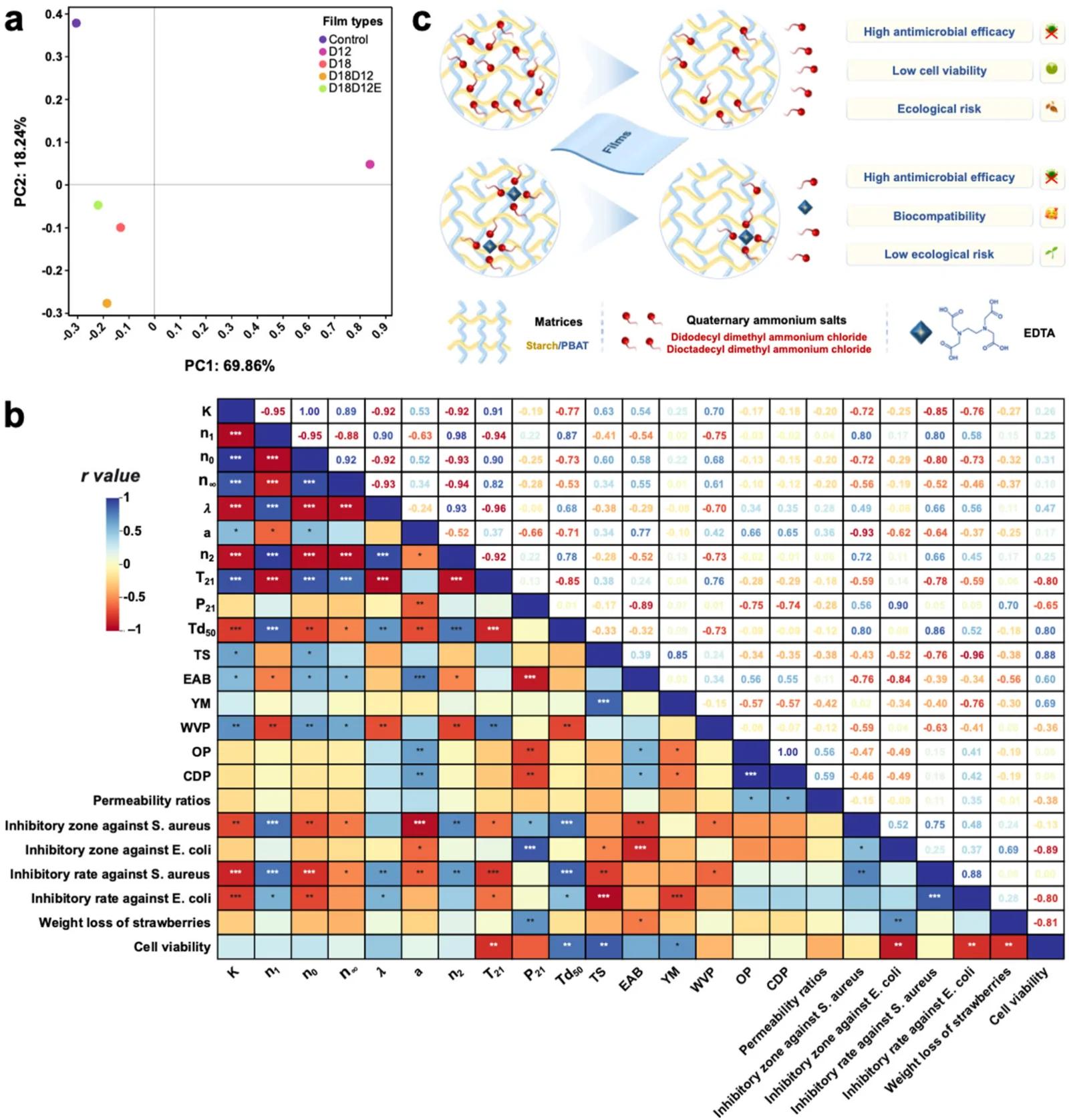
(**a**) PCA plot of the film formulations based on their physicochemical and functional properties. (**b**) Inter-group correlation heatmap of the film properties. (**c**) Schematic illustration summarizing the proposed role of QASs and EDTA in regulating the balance between antibacterial efficacy and preliminary biological safety. *, **, and *** indicate significant correlations at *p* < 0.05, *p* < 0.01, and *p* < 0.001, respectively.

Principal component analysis (PCA) was performed to evaluate the overall differences among the composite films based on their physicochemical and functional properties (Figure 7a). PC1 and PC2 explained 69.86% and 18.24% of the total variance, respectively, indicating that these two principal components captured the major variation in the dataset [54]. The control film was clearly separated from the QAS- and EDTA-containing films, suggesting that their incorporation altered the comprehensive performance profile of the resulting films. In particular, D12 was positioned far from the other modified films along PC1, while D18, D18D12, and D18D12E were distributed on the negative side of PC1, reflecting the different contributions of alkyl-chain length, mixed QAS, and EDTA incorporation to film structure and functionality.

The correlation heatmap in Figure 7b further illustrates the relationships among rheological behavior, moisture mobility, thermal stability, mechanical properties, barrier performance, antibacterial activity, and cell viability. Rheological parameters showed close associations with mechanical and barrier performance, suggesting that melt flow behavior and chain mobility contributed to the final film properties. Gas permeability parameters, including OP and CDP, were strongly correlated with each other, reflecting similar diffusion behavior of those non-polar gases through the matrix. Antibacterial indices were also correlated with some structural and rheological parameters, implying that the accessibility and distribution of QAS functional groups within the composite matrix may affect antibacterial efficacy. Notably, cell viability showed opposite trends to some antibacterial-related indicators, suggesting a potential trade-off between antibacterial activity and biological safety. As shown in Figure 7c, QASs introduce cationic antibacterial functionality into the starch/PBAT matrix, accompanied by reduced cell compatibility when the balance between functional group exposure and matrix restriction is not well controlled. The further introduction of EDTA may regulate intermolecular interactions and matrix packing through hydrogen bonding with starch hydroxyl groups, maintaining antibacterial efficacy while reducing potential biological risk.

## 4. Conclusions

In this study, starch/PBAT antibacterial films containing QASs and EDTA were prepared by extrusion blowing. The incorporation of EDTA enabled the D18D12E film to achieve antibacterial rates of ≥99% against both *S. aureus* and *E. coli*, while reducing the total QAS loading by one-third. Compared with the control, its tensile strength decreased from 11.62 to 7.60 MPa, whereas the oxygen and carbon dioxide permeability coefficients increased from 13.26 and 12.82 to 22.42 and 22.10 × 10^−13^ cm^3^·cm·cm^−2^·s^−1^·Pa^−1^, respectively. Nevertheless, the film maintained a high elongation of 805.80% and a water vapor permeability of 4.03 × 10^−11^ g·m·m^−2^·s^−1^·Pa^−1^, neither of which differed significantly from the control. In the storage tests, D18D12E delayed the visible deterioration of strawberries for up to 8 days and Chinese steamed bread for 4 days. The film also showed a HepG2 cell viability of 75.4%, did not prevent tobacco seeds from germinating and developing into seedlings in film-containing soil, and exhibited overall migration below 10 mg/dm^2^ under the tested conditions. Overall, partial replacement of QASs with EDTA maintained strong antibacterial activity at a lower QAS loading.

Nevertheless, several limitations should be considered. The storage assessment relied mainly on visual observations without food microbial counts, compound-specific migration was not measured, and the biological controls and replication of some instrumental analyses, including TG, were limited. Future work should incorporate quantitative food-quality measurements, targeted migration analyses, broader biological models, etc.

## Figures and Tables

**Table 1:** Sample codes and formulations of the starch/PBAT films.

Sample Codes	Starch (g)	PBAT (g)	Glycerol (g)	DK3 (g)	D12 (g)	D18 (g)	EDTA (g)
Control	400	600	140	50	-	-	-
D12	400	600	140	50	30	-	-
D18	400	600	140	50	-	30	-
D18D12	400	600	140	50	15	15	-
D18D12E	400	600	140	50	10	10	10

**Table 2:** Rheological fitting results based on the Power-law and Carreau–Yasuda models.

Samples	Power-Law Model		Carreau–Yasuda Model				
	*K*	*n*_1_	η_0_	η_∞_	*λ*	*a*	*n*_2_
Control	14,240.00	0.35	24,602.60	611.71	1.66	138.54	0.16
D12	6714.60	0.44	12,739.33	397.83	2.22	46.14	0.28
D18	7288.20	0.47	14,193.94	420.24	2.48	51.21	0.33
D18D12	4200.39	0.48	8939.13	224.17	3.09	113.71	0.36
D18D12E	9883.71	0.40	18,966.88	626.36	2.02	108.48	0.19

## Data Availability

The original contributions presented in this study are included in the article/Supplementary Materials. Further inquiries can be directed to the corresponding author.

## Supplementary Materials

The following supporting information can be downloaded at: https://www.mdpi.com/article/10.3390/foods15162797/s1. Figure S1. Cross-sectional SEM image of the D18D12 film at 5000× magnification. Yellow arrows indicate microcracks adjacent to the granular features. Table S1. Thermal degradation parameters of the starch/PBAT composite films. Table S2. Two-factor statistical analysis for weight loss rates.
