# Supplementary material for: Starch/Poly(butylene adipate-co-terephthalate) Blown Films Containing Quaternary Ammonium Salts and Ethylenediaminetetraacetic Acid for Food Preservation
Source: Foods. 2026 Aug 10;15(16):2797. doi: 10.3390/foods15162797 (PMC13512301; doi:10.3390/foods15162797)
Supplement: Supplementary file 1 [file foods-15-02797-s001.zip › foods-4421569-supplementary.pdf]

Supplementary Figure S1 Cross-sectional SEM image of the D18D12 film at 5000× magnification.

Yellow arrows indicate microcracks adjacent to the granular features

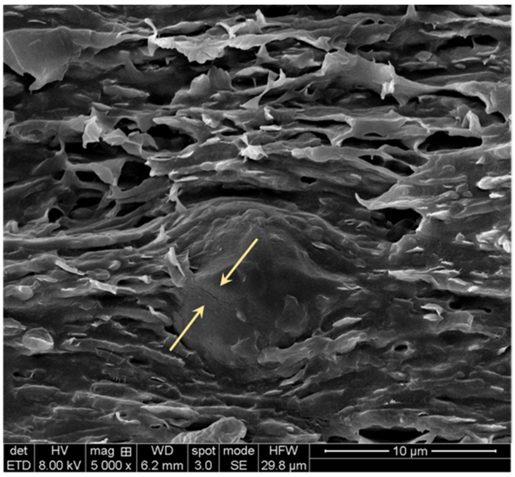

Supplementary Table S1 Thermal degradation parameters of the starch/PBAT composite films.

Sample	T_{d5} (°C)	T_{d10} (°C)	T_{d50} (°C)	Residual mass at 600 °C (%)
Control	169	209	343	10.51
D18	161	196	348	8.95
D12	164	201	352	8.84
D18D12	168	204	349	9.16
D18D12E	165	199	348	9.90

Values were obtained from one thermogravimetric run for each formulation. T_{d5}, T_{d10}, and T_{d50} denote the temperatures corresponding to mass losses of 5%, 10%, and 50%, respectively, and were obtained from the nearest experimental data points on the corresponding TG curves. Residual mass was determined at approximately 600 °C.

Supplementary Table S2 Two-factor statistical analysis for weight loss rates.

Packaging type	Storage time (d)				*P* value of Packaging type × Storage time
	2	4	6	8	
Unpackaged	7.99^{Ad}	18.91^{Ac}	38.76^{Ab}	50.59^{Aa}	
PE	0.37^{Ca}	0.63^{Ca}	0.95^{Da}	1.87^{Ca}	
Control	2.59^{Bb}	3.36^{Bb}	5.21^{Ba}	6.37^{Ba}	
D18	2.43^{Bb}	2.98^{Bb}	5.19^{BCa}	5.89^{Ba}	***
D12	2.77^{Bc}	3.43^{Bc}	5.33^{Bb}	7.39^{Ba}	
D18D12	2.52^{Bb}	3.65^{Bb}	5.59^{Ba}	6.63^{Ba}	
D18D12E	2.21^{Bb}	2.87^{Bb}	3.54^{Cb}	5.94^{Ba}	

Results are quoted as means of triplicate determinations. a–c: Means in the rows with different letters differ as $P < 0.05$. A–D: Means in the columns with different letters differ as $P < 0.05$. Significance of *P*-value: ***, $P < 0.001$; **, $P < 0.01$; *, $P < 0.05$; NS, $P > 0.05$ (non-significant).
